# *S*-Ketamine Oral Thin Film—Part 1: Population Pharmacokinetics of *S*-Ketamine, *S*-Norketamine and *S*-Hydroxynorketamine

**DOI:** 10.3389/fpain.2022.946486

**Published:** 2022-07-11

**Authors:** Pieter Simons, Erik Olofsen, Monique van Velzen, Maarten van Lemmen, René Mooren, Tom van Dasselaar, Patrick Mohr, Florian Hammes, Rutger van der Schrier, Marieke Niesters, Albert Dahan

**Affiliations:** ^1^Department of Anesthesiology, Leiden University Medical Center, Leiden, Netherlands; ^2^LTS Lohmann Therapie-Systeme AG, Andernach, Germany; ^3^PainLess Foundation, Leiden, Netherlands

**Keywords:** ketamine, modeling, pharmacokinetics, neuropathic pain, oral thin film, *S*-ketamine

## Abstract

Ketamine is administered predominantly *via* the intravenous route for the various indications, including anesthesia, pain relief and treatment of depression. Here we report on the pharmacokinetics of sublingual and buccal fast-dissolving oral-thin-films that contain 50 mg of *S*-ketamine in a population of healthy male and female volunteers. Twenty volunteers received one or two oral thin films on separate occasions in a randomized crossover design. The oral thin films were placed sublingually (*n* = 15) or buccally (*n* = 5) and left to dissolve for 10 min in the mouth during which the subjects were not allowed to swallow. For 6 subsequent hours, pharmacokinetic blood samples were obtained after which 20 mg *S*-ketamine was infused intravenously and blood sampling continued for another 2-hours. A population pharmacokinetic analysis was performed in NONMEM pharmacokinetic model of *S*-ketamine and its metabolites *S*-norketamine and *S*-hydroxynorketamine; *p* < 0.01 were considered significant. *S*-ketamine bioavailability was 26 ± 1% (estimate ± standard error of the estimate) with a 20% lower bioavailability of the 100 mg oral thin film relative to the 50 mg film, although this difference did not reach the level of significance. Due to the large first pass-effect, 80% of *S*-ketamine was metabolized into *S*-norketamine leading to high plasma levels of *S*-norketamine following the oral thin film application with 56% of *S*-ketamine finally metabolized into *S*-hydroxynorketamine. No differences in pharmacokinetics were observed for the sublingual and buccal administration routes. The *S*-ketamine oral thin film is a safe and practical alternative to intravenous *S*-ketamine administration that results in relatively high plasma levels of *S*-ketamine and its two metabolites.

## Introduction

Over the last decade, low-dose ketamine has gained in popularity for treatment of chronic pain and therapy-resistant depression (1). Since its discovery in the early 1960s, ketamine has been administered mostly *via* the parenteral route for the induction of anesthesia and procedural sedation. With a broader range of indications and pre-hospital and out-of-hospital use of ketamine, the need for skilled venipunctures is a hurdle for chronic and repeated ketamine administrations. To overcome this problem different routes of ketamine administration have been studied extensively, including inhaled, oral, sublingual, nasal, subcutaneous, intramuscular and rectal administrations. All of these routes have advantages, such as simplicity of administration, and drawbacks. For example, oral dosing results in slow absorption and is largely subject to intestinal and first-pass metabolism, with unpredictable bioavailability (7–25%) (2). Others, such as the subcutaneous or intramuscular administration routes, are invasive and also result in a relatively slow absorption (2, 3). Here we study the pharmacokinetics (and in part 2 of this study (4), the pharmacodynamics) of sublingual and buccal fast-dissolving oral-thin-films (OTFs) that contain 50 mg of *S*-ketamine, one of the stereoisomers of ketamine. In this report, we present the results of a pharmacokinetic analysis of the concentration-time curves following sublingual or buccal administration of 50 or 100 mg *S*-ketamine OTF in healthy volunteers. Apart from the simplicity in application, the use of an *S*-ketamine OTF may, depending on its bioavailability and first-pass effect, be advantageous in the treatment of pain and depression. An acceptable level of *S*-ketamine bioavailability will make it suitable for pain treatment in an acute setting (2, 5), while a large first-pass effect with high concentrations of hydroxynorketamine will make the *S*-ketamine OTF an interesting alternative for management of therapy-resistant depression as there is evidence that this metabolite is a potent antidepressant (6, 7). We performed a population pharmacokinetic analysis of the *S*-ketamine OTF in healthy volunteers, and considered the parent compound and its metabolites, *S*-norketamine and *S*-hydroxynorketamine in the analysis.

## Methods

### Ethics and Subjects

The protocol was approved by the Central Committee on Research Involving Human Subjects (Competent authority: Centrale Commissie Mensgebonden Onderzoek (CCMO), The Hague, the Netherlands; registration number NL75727.058.20) and the Medical Research Ethics Committee at Leiden University Medical Center (Medische Ethische Toetsingscommissie Leiden-Den Haag-Delft, the Netherlands; identification number P20.111). The study was registered at the trial register of the Dutch Cochrane Center (www.trialregister.nl) under identifier NL9267 and at the European Union Drug Regulating Authorities Clinical Trials (EudraCT) database under number 2020-005185-33. All procedures were performed in compliance with the latest version of the Declaration of Helsinki and followed Good Clinical Practice guidelines.

Healthy male and female volunteers, aged 18–45 years and with a body mass index ≥ 19 kg/m^2^ and ≤ 30 kg/m^2^, were recruited. After recruitment, all subjects gave written and oral informed consent, after which they were screened. Additional inclusion criteria were ability to communicate with the research staff, non-smoking for at least 3 months prior to screening, and deemed suitable by the investigators. Exclusion criteria included: presence or history of any medical or psychiatric disorder (including a history of substance abuse, anxiety or a chronic pain syndrome), use of medication in the 3 months prior to screening (including vitamins and herbs, excluding oral contraceptives), use of more than 21 units of alcohol per week, use of illicit substances (including cannabis) in the 4 weeks prior to the study, a positive urine drug test or an alcohol breath test at screening or on the morning of test drug dosing, pregnancy, lactating or a positive pregnancy test at screening or on the morning of dosing, participation in another (drug) trial in the 60 days prior to dosing. Eating, drinking, tooth brushing or gum chewing was not allowed on the morning of oral thin film application to avoid changes/variabilities in saliva pH, which could potentially affect the mucosal permeability and *S*-ketamine uptake.

### Study Design

#### *S*-Ketamine Oral Thin Film Placement—Randomization—Intravenous *S*-Ketamine Infusion

This phase 1 study had an open-label randomized crossover design. The subjects were randomized to receive one oral thin film on one occasion (50 mg *S*-ketamine) and two on another visit (100 mg *S*-ketamine) with at least 7 days between visits. The thin film is a rectangular 4.5 cm^2^ orodispersible film containing 57.7 mg *S*-ketamine hydrochloride (*S*-ketamine HCL). The *S*-ketamine HCL is dispersed within a matrix to produce a film corresponding to 50 mg *S*-ketamine free base. The film(s) was/were placed either under the tongue or buccally on the mucosa. After placement of the films, the subject was not allowed to swallow for 10 min. The randomization sequence was determined by the randomization option in the Electronic Data Capture system CASTOR (www.castoredc.com). The oral thin films were provided by LTS Lohmann Therapie-Systeme AG (Andernach, Germany) and were dispensed by the hospital trial pharmacy on the morning of dosing. To calculate the bioavailability of the OTF, six hours after placement of the oral thin film, all subjects received an intravenous *S*-ketamine (Ketanest-S, Pfizer, the Netherland) infusion of 20 mg over 20 min. The intravenous dose of 20 mg given was based on a previous study on the pharmacokinetics of inhaled *S*-ketamine in which a 20 mg intravenous dose was administered over 20 min. This dose was well accepted by the volunteers (8). We waited 6 hours before giving the intravenous dose to ensure that most of the pharmacodynamic effects (*i.e*. the topic of our accompanying paper) (4) had dissipated.

#### Blood Sampling and *S*-Ketamine Measurement

Blood samples were obtained at t = 0 (= oral thin film placement) 5, 10, 20, 40, 60, 90, 120, 180, 240, 300, 360 min, and at the following time periods following the start of the intravenous administration: 2, 4, 10, 15, 20, 30, 40, 60, 75, 90, and 120 min. 3-mL samples were obtained from a 22G arterial line placed in the radial artery of the non-dominant arm and collected in lithium heparin tubes. All heparin samples were centrifuged at 1,500 g for 10 min, within 15 min after withdrawal and plasma was separated and stored in two aliquots at −80°C until analysis.

For analysis the samples were thawed and 200 μL was transferred into glass tubes and 10 μL internal standard was added. After again mixing, 4 mL methyl-tertiair-butylether was added, followed by 15 min centrifugation. The upper organic layer was pipetted into another tube that contained 0.6 mL of 0.4 mol/L hydrochloric acid in methanol, and dried under a gentle stream of nitrogen at 35°C. The residue was re-dissolved in 100 μL mobile phase (6.8 % methanol in water with 0.1 % formic acid) by vortexing and ultrasonication for 3 min and 5 μL sample was injected on the chromatographic system with a C18 column.

All reference standards (ketamine and norketamine) and internal standards ketamine-D4 (K-D4), norketamine-D4 (NK-D4) were HCl salts and purchased from LGC Standards GmbH (Germany); cis-6-hydroxynorketamine (6-HNK) was purchased from Syncom BV (the Netherlands); and the internal standard hydroxynorketamine- ^13^C6 (HNK-13C6) was purchased from Alsachim SAS (France).

*S*-ketamine and its metabolites, *S*-norketamine and *S*-hydroxynorketamine were measured at the Department of Pharmacy and Toxicology and Center for Proteomics and Metabolomics, both at LUMC, according to EMA guideliness, using liquid chromatography coupled to QTOF-MS (hybrid quadrupole time-of-flight mass spectrometry) as detection technique (i.e., LC-QTOF-MS/MS). The LC-QTOF-MS/MS system consisted of a Thermo Scientific double pump 3,000 gradient system gradient with Bruker IL-2 QTOF.A column (Xterra MS C18 3.5 μm x 2.1 mm x 100 mm) and precolumn (Xterra MS C18 Vanguard cartridge 3.5 μm x 2.1 mm) and was purchased from Waters Chromatography Europe BV (the Netherlands). For separation the mobile phase was methanol/water with 0.1 % formic acid with a gradient from 6.8 to 96 % methanol from 1 until 8.5 min. The total separation time was 15 min with a flow rate of 0.3 ml/min. The eluent was directed to the QTOF-MS from 1.2 until 7 min while the other part was directed to waste by a valve to avoid contamination of the QTOF. The system was controlled by Chromeleon Chromatography Data System software (Thermo Fisher Scientific, the Netherlands) for the LC part and Hystar (Bruker Nederland BV, the Netherlands) for the QTOF/MS part. In the positive ionization mode, the masses of the M+H ions were respectively 224.084, 228.109, 238.0993, 242, 124, 240.0786 and 246.099 Da for norketamine, norketamine-D4, ketamine, ketamine-D4, Cis-6-hydroxynorketamine and hydroxynorketamine-^13^C6.

Quant Analysis (Bruker Nederland BV, the Netherlands) was used for quantification of all analytes with a weighed (1/X^*^X) calibration line. The lower limits of quantitation were 6 ng/ml (0.025 nmol/mL), 6 ng/ml (0.026 nmol/mL) and 4 ng/ml (0.01 nmol/mL), for *S*-ketamine, *S*-norketamine and *S*-hydroxynorketamine, respectively. The upper limits of quantitation were, respectively, 500 ng/ml (2.1 nmol/ml), 1,000 (4.4 nmol/ml) and 200 ng/mL (0.72 nmol/ml) for *S*-ketamine, *S*-norketamine and *S*-hydroxynorketamine.

#### Adverse Events

Reported adverse events related to treatment were collected and were split up into events related to the 50 or 100 mg oral thin film or to the intravenous administration of *S*-ketamine. Additionally, the subjects were queried for dissociative side effects using the Bowdle questionnaire (9). The Bowdle questionnaire allows derivation of three factors of psychedelic ketamine effects: drug high and changes in internal and external perception. All three were measured on a visual analog score from 0 (no effect) to 10 cm (maximum effect). The questionnaire was first published in 1998 as a hallucinogen rating scale to quantify ketamine-induced psychedelic symptoms in volunteers and has been used in multiple studies on the effect of various psychedelics on dissociative symptoms. Blood pressure was obtained from the arterial-line using the FloTrac and Hemosphere system (Edwards Lifesciences, Irvine USA).

### Population Pharmacokinetic Analysis

Data analysis was performed using NONMEM version 7.5.0 (ICON Development Solutions, Hanover, MD, USA). To account for the differences in molecular weight between *S*-ketamine and the metabolites, concentration data were converted from ng/ml to nmol/ml. Data were analyzed in a stepwise fashion. First, *S*-ketamine data were analyzed, followed by the addition of *S*-norketamine and subsequently *S*-hydroxynorketamine. The routing of *S*-ketamine consists of two parts: one direct pathway from the OTF into plasma, and one indirect pathway in which some *S*-ketamine is stored in saliva which is ingested and absorbed *via* the gastrointestinal tract. Since *S*-norketamine was not administered, theoretically, the volume of the central *S*-norketamine compartment (VN1) was not identifiable. However, since we assumed that 80% of *S*-ketamine was metabolized VN1 is identifiable (2). The same applies for *S*-hydroxynorketamine: since we assumed that 70% of *S*-norketamine is transformed into *S*-hydroxynorketamine, (10) the volume of the central *S*-hydroxynorketamine compartment is identifiable. The number of *S*-ketamine, *S*-norketamine and *S*-hydroxynorketamine compartments as well as the intermediary metabolism compartments was determined by goodness-of-fit criteria, i.e., a significant decrease in objective function value (OFV) calculated as −2 log likelihood (χ^2^ test), visual inspection of the data fits and goodness-of-fit plots (normalized prediction distribution error vs. time plots, normalized prediction distribution error vs. predicted plots, and predicted vs. measured plots). Moreover, prediction-variance-corrected visual predictive checks (VPCs) were performed by simulating 1,000 data sets based on the model parameters and comparing the simulated quantiles with those of the true data. *P* < 0.01 were considered significant.

FOCE-I (first-order conditional estimation with interaction) was used to estimate model parameters. To account for inter-individual and inter-occasion variability (IOV), random effects were included in the model with an exponential relation: θ_i_ = θ × exp(η_i_ + η_iov_), where θ_i_ is the parameter for individual i, θ is the population parameter, η_i_ is the random difference between the population and individual parameter, and η_iov_ is the difference between θ_i_ and θ as a result of IOV. In addition, proportional and additive errors were evaluated for each separate analyte to account for residual variability. The proportional and combined proportional and additive error models were described by Y_*ij*_ = P_*ij*_ × (1 + ε_*ij*_) and Y_*ij*_ = P_*ij*_ × (1 + ε_1ij_) + ε_2ij_, respectively, where Y_*ij*_ is the jth observed plasma concentration for individual i, P_*ij*_ is the corresponding model prediction, and ε_ij_ is the residual error. Inter-occasion variability was determined for the *S*-ketamine and *S*-norketamine absorption parameters, while it was determined for all *S*-hydroxynorketamine model parameters.

### Simulations

*In-silico* simulations were performed to determine the effect of changes in the duration that the 50 mg *S*-ketamine oral thin film stayed sublingually (before the subjects was allowed to swallow) on plasma concentrations of *S*-ketamine and its metabolites. To that end, factor D1 was either increased or decreased by a factor (F) of 2, F1 was adjusted assuming it converges to 1 exponentially with D1 (i.e., F1 approaches 1 in case the OTF remains sublingually and is not swallowed), F2 was adjusted so that total bioavailability remains constant, and changes in D2 followed changes in D1 assuming D2 is the sum of D1 and gastrointestinal lag times. D1 is the duration of basorption, D2 is duration of absorption form the gastrointestinal tract. F1 and F2 are the *S*-ketamine bioavailability from the oral mucosa and gastrointestinal tract, respectively.

## Results

**Figure 1:**
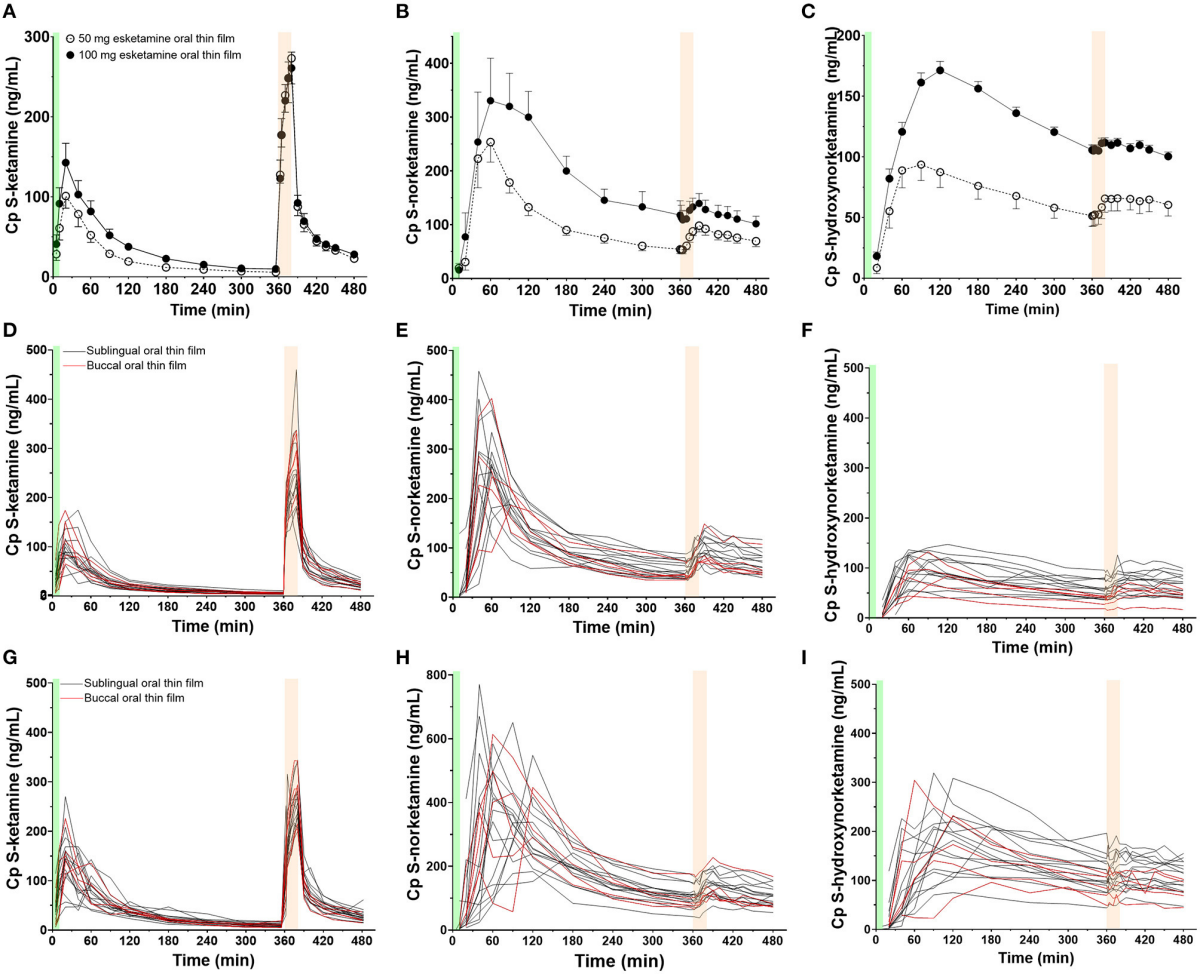
Mean measured plasma concentrations following application of the 50 and 100 mg *S-k*etamine oral thin film (OTF): **(A)** *S*-ketamine, **(B)** *S*-norketamine, and **(C)** *S*-hydroxynorketamine. Individual concentrations are given in panels **(D–F)** for the 50 mg oral thin film and **(G–I)** for the 100 mg oral thin film. In black the results of placement below the tongue, in red buccal placement. The OTF was administered at t = 0 min for 10 min (green bars); at t = 360 min, an intravenous dose of 20 mg *S*-ketamine was administered over 20 min (light orange bars).

Twenty-three subjects were screened, of which three subjects were excluded from participation because of psychological issues (*n* = 2) or earlier alcohol abuse (*n* = 1). Twenty subjects were dosed at least once (see Table 1 for their demographic characteristics), 19 subjects were dosed twice (once OTF with 50 mg *S*-ketamine, once with 100 mg *S*-ketamine). One subject declined further participation after completing the first session, receiving 100 mg *S*-ketamine OTF sublingually, due to psychotomimetic side effects that occurred during the intravenous *S*-ketamine infusion. The mean and individual *S*-ketamine, *S*-norketamine and *S*-hydroxynorketamine data for both the sublingual and buccal OTF and intravenous infusion are given in Figure 1. Since no differences were observed in plasma concentrations for the sublingual (*n* = 15) or buccal (*n* =5) locations of the OTF (individual data in Figure 1 panels D-I with in red buccal administration and in black sublingual administration) and in the subject characteristics (Table 1), we merged the two subgroups in the pharmacokinetic model analyses. Peak concentration (CMAX), time of peak concentration (TMAX) and area-under-the-concentration-time curves (AUC) of *S*-ketamine and its metabolites are given in Table 2. These data indicate that increasing the *S*-ketamine OTF dose produces dose a dependent increase in CMAX for *S*-ketamine and its metabolites, with a delay in CMAX for the downstream metabolites. Comparing these data to the values observed after the intravenous *S*-ketamine in Figures 1A–C, administration indicate the greater metabolism of the *S*-ketamine from the OTF compared to the 20 mg intravenous *S*-ketamine. Peak *S*-ketamine concentrations after the intravenous infusion were 273 (259–287) ng/mL [mean (95% confidence interval)] after treatment with the 50 mg *S*-ketamine OTF and 260 (251–269) ng/mL after treatment with the 100 mg *S*-ketamine OTF (Figure 1).

**Table 1:** Subject characteristics.

	**Total population; *n* = 20**	**Sublingual OTF; *n* = 15**	**Buccal OTF; *n* = 5**
Age (yrs) ± SD (range)	24 ± 3 (19–32)	24 ± 3 (21–30)	25 ± 5 (19–32)
Sex (M/F n)	10/10	8/7	2/3
Mean weight (kg) ± SD (range)	73 ± 12 (53–93)	72 ± 13 (53–93)	74 ± 8 (64–85)
Mean height (cm) ± SD (range)	179 ± 10 (161–197)	179 ± 12 (161–197)	177 ± 6 (170–183)
Mean BMI (kg/m^2^) ± SD (range)	23 ± 2 (19–27)	22 ± 2 (19–27)	24 ± 3 (21–27)

*BMI = body mass index*.

**Table 2:** Peak concentration (CMAX), time of CMAX, and area-under-the time-concentration curve (AUC) of *S*-ketamine, *S*-norketamine and *S*-hydroxynorketamine following 50 and 100 mg *S*-ketamine oral thin film (OTF).

	**50 mg *S*-ketamine OTF**	**100 mg *S*-ketamine OTF**
	***S*** **-ketamine**	
CMAX (ng/ml)	96 (81–111)	144 (127–161)
CMAX (nM)	420 (360–480)	600 (500–700)
Tmax (min)	18.8 (16.6–21.2)	19.1 (17.1–21.2)
AUC (0-6 h) (ng/ml.min)	8,363 (7,263–9464)	13,347 (11,933–14,760)
	***S*** **-norketamine**	
CMAX (ng/ml)	276 (243–308)	426 (362–489)
CMAX (nM)	1,130 (970–1,300)	1475 (1,122–2,237)
Tmax (min)	61 (53–68)	78 (66–91)
AUC (0–6 h) (ng/ml.min)	38,497 (34,131–42,863)	67,959 (60,045–75,872)
	***S*** **-hydroxynorketamine**	
CMAX (ng/ml)	101 (89–115)	189 (160–218)
CMAX (nM)	340 (293–387)	619 (594–644)
Tmax (min)	81 (69–92)	109 (89–130)
AUC (0–6 h) (ng/ml.min)	24,087 (20,694–27,480)	44,972 (38,563–51,382)

*Values are mean (95% confidence interval)*.

### Adverse Events

Eighteen subjects reported at least one adverse event. In total there were 97 adverse events. None were serious adverse events. See for prevalence of events Table 3. We just relate one adverse event (numbness of the tongue) directly to the application of the oral thin film, the remaining events were drug associated. All subjects experienced dissociative side effects (drug high, changes in internal and external perception) as derived from the Bowdle questionnaire. These data are presented in detail in the accompanying paper on the OTF pharmacodynamic effects (4). During the first hour after application of the OTF, blood pressure increased with mean arterial pressure 92 ± 11 mmHg (mean ± SD), 97 ± 7 mmHg and 104 ± 6 mmHg at baseline (prior to application) and 10 and 60 min after the application of the 50 mg *S*-ketamine OTF, respectively, and 95 ± 15 mmHg, 97 ± 11 mmHg and 108 ± 10 mmHg at baseline and 10 and 60 min after the application of the 100 mg *S*-ketamine OTF.

**Table 3:** Adverse effects.

	**50 mg**	**100 mg**	**20 mg intravenous**
	***S*-ketamine OTF**	***S*-ketamine OTF**	***S*-ketamine**
Blurred vision	1		
Feeling drunk	2		
Bradykinesia	1	1	
Whistling sound in the ears			1
Vertigo/dizziness	1	3	4
Drowsiness			3
Nausea	1	1	2
Headache	1	2	3
Numbness of the tongue		2	
Hypertension (SBP > 180 mmHg)			2
Perspiration			1
Dry eyes			1
Dissociative effects^*^	20	20	20
Total	27	29	37

* *Dissociative effects included drug high and changes in internal and external perception*.

### Population Pharmacokinetic Analysis

**Figure 2:**
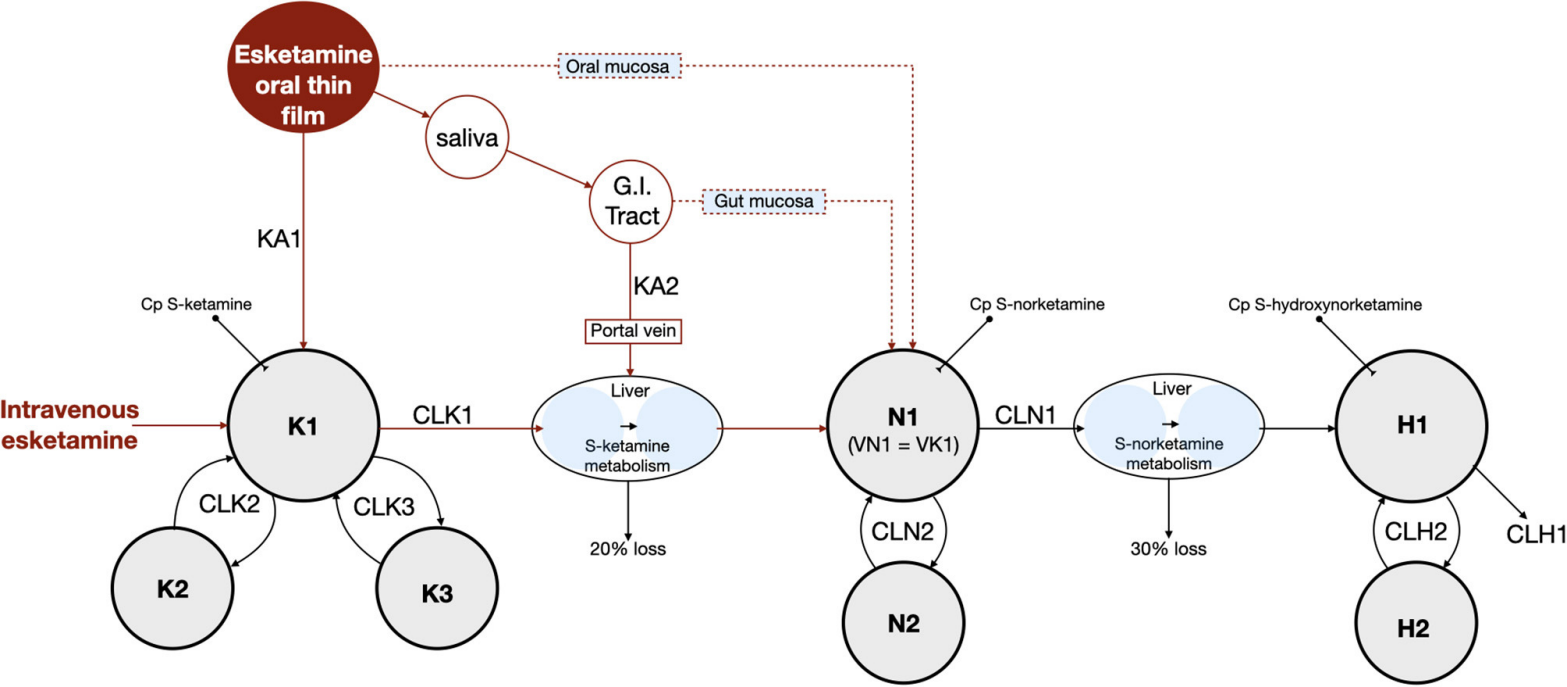
Final pharmacokinetic model. K = *S*-ketamine, N = *S*-norketamine and H = *S*-hydroxynorketamine. KA1 and KA2 are *S*-ketamine rate constants. G.I. tract = gastrointestinal tract. Cp = plasma concentration. K1, N1 and H1 are the central compartments for *S*-ketamine, *S*-norketamine and *S*-hydroxynorketamine, respectively. VN1 and VK1 are the volumes of the central compartments of *S*-ketamine and *S*-norketamine, respectively. Kx, Nx and Hx are the peripheral compartments for *S*-ketamine, *S*-norketamine and *S*-hydroxynorketamine, respectively, with x = compartment 2 or 3. CL = clearance with CLK1 and CLN1 *S*-ketamine and S-norketamine clearances from the central compartment toward the metabolism compartment, respectively and CLK2, CLK3, CLN2 and CLH2 intercompartmental clearances. CLH1 is the terminal *S*-hydroxynorketamine clearance. MTT = mean transit (or delay) time with MTTG the mean transit time from the gut to the liver.

**Figure 3:**
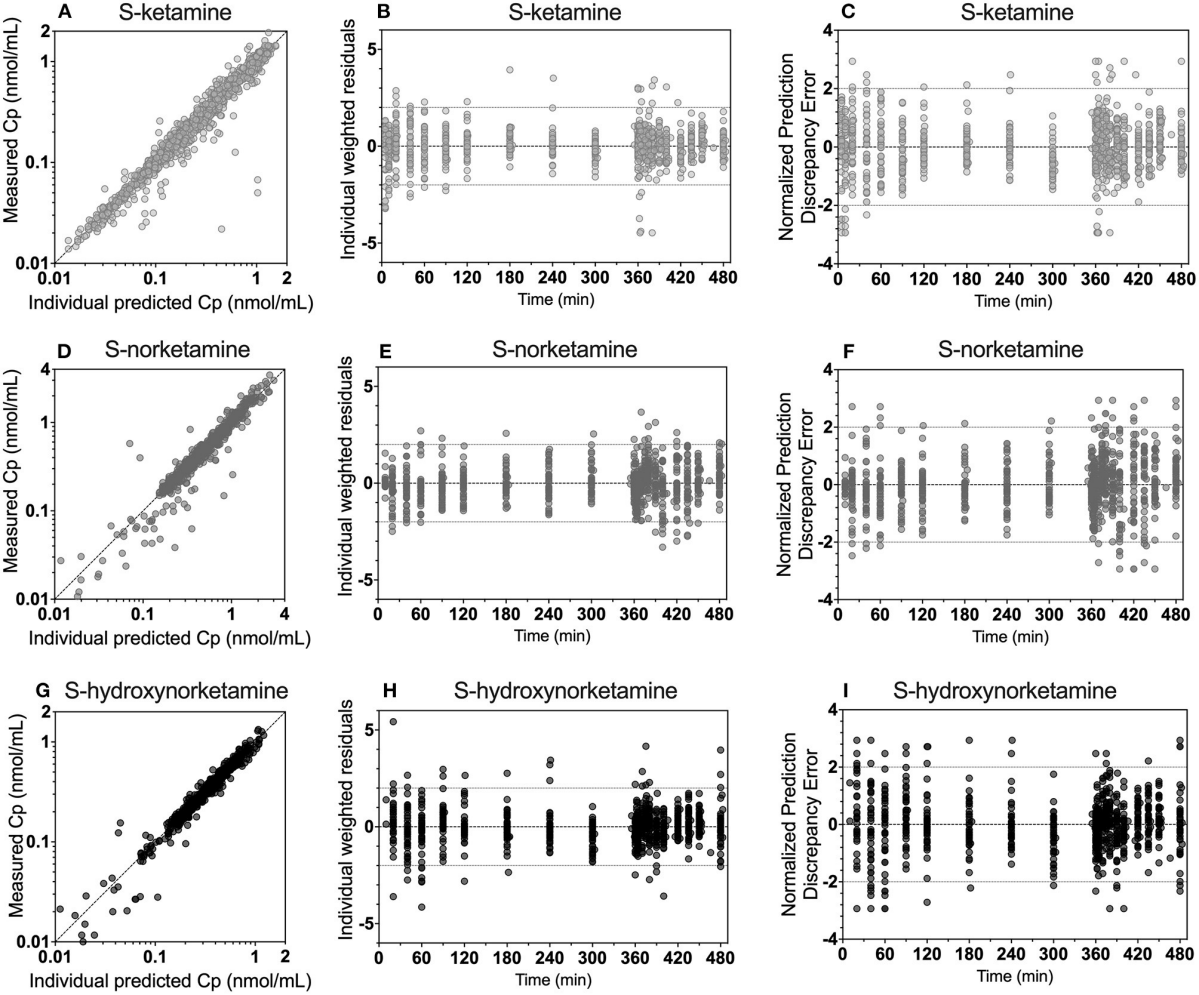
Goodness-of-fit plots for *S*-ketamine **(A–C)**, *S*-norketamine **(D–F)** and *S*-hydroxynorketamine **(G–I)**. **(A**,**D,G)**: measured concentration vs. individual predicted. **(B,E,H)**: individual weighted residuals vs. time. **(C,F,I)**: Normalized discrepancy errors vs. time.

The schematic diagram of the final pharmacokinetic model of the absorption of *S*-ketamine from the OTF and disposition of *S*-ketamine, with three compartments, and its metabolites *S*-norketamine and *S*-hydroxynorketamine, with each two compartments, is given in Figure 2. Model parameter estimates are given in Table 4; *S*-ketamine and *S*-norketamine distribution- and clearance-related parameters are in close correspondence with earlier data derived from a pooled-analysis of data from the literature (11). Gastrointestinal absorption of *S*-ketamine and the metabolism of *S*-ketamine and *S*-norketamine were best described by two delay or metabolism compartments. The model parameters given in Table 4 are explained in Figure 2. All pharmacokinetic data fits are presented in Supplementary Figure 1; the goodness-of-fit plots (individual predicted concentration vs. measured concentration; individual weighted residuals over time; normalized prediction discrepancy errors) are given in Figure 3. Inspection of these plots together with the individual data fits indicate that the final model adequately described the plasma concentration-time data of *S*-ketamine and its two measured metabolites.

**Table 4:** *S*-ketamine OTF pharmacokinetics.

**Parameter**	**Estimate**	**SEE**	**Inter-subject variability (ω^2^)**	**SEE**	**Inter-occasion variability (ν^2^)**	**SEE**
***S*** **-ketamine mucosal absorption from OTF**						
F1 (bioavailability) %	26.3	1.2			0.060	0.019
D1 (duration of absorption) min	13.1	1.0			0.154	0.033
Absorption rate constant; KA1 (min^−1^)	0.04	0.002			0.062	0.014
Outlier (id = 4, occ = 2) for KA1 (min^−1^)	0.012	0.0004				
Volume of *S*-ketamine compartment 1; VK1 (L @ 70 kg)	11.6	0.9	0.057	0.019		
Volume of *S*-ketamine compartment 2; VK2 (L @ 70 kg)	39.0	2.9				
Volume of *S*-ketamine compartment 3; VK3 (L @ 70 kg)	174	11				
Clearance from VK1 toward metabolism compartment MK; CLK1 (L/min @ 70 kg)	1.48	0.06	0.029	0.012		
Clearance from VK1 to VK2; CLK2 (L/min @ 70 kg)	2.43	0.24				
Clearance from VK1 to VK3; CLK3 (L/min @ 70 kg)	1.21	0.08	0.026	0.014		
σ_Relative_ (relative within subject variability)	0.012	0.0004				
***S*** **-ketamine absorption from the gastrointestinal tract**						
F2 (bioavailability) %	116	6			0.057	0.031
D2 (duration of infusion) min	29.9	3.5			0.611	0.120
Absorption rate constant; KA2 (min^−1^)	0.049	0.007			0.376	0.150
Mean transit time GUT (min)	10.7	1.7			0.937	0.312
						
***S*** **-norketamine**						
Volume of *S*-norketamine compartment 1; VN1	11.6	0.9	0.057	0.019	11.6	
Volume of *S*-norketamine compartment 2; VN2 (L @ 70 kg)	221	13				
Clearance of *S*-norketamine compartment 1; CLN1 (L/min @ 70 kg)	1.00	0.04	0.050	0.012		
Clearance of *S*-norketamine compartment 2; CLN2 (L/min @ 70 kg)	2.63	0.15				
Mean transit time K → NK (min)	20.1	1.0	0.021	0.122		
Outlier mean transit time (id = 4) (min)	8.72	0.19				
σ_Relative_ (relative within-subject variability)	0.102	0.007				
σ_Additive_ (additive within-subject variability)	0.058	0.018			0.751	0.349
						
***S-*** **hydroxynorketamine**						
Volume of *S*-hydroxynorketamine compartment 1; VH1 (L @ 70 kg)	4.4	2.0	1.22	0.91		
Volume of *S*-hydroxynorketamine compartment 2; VH2 (L @ 70 kg)	87.5	6.5			0.152	0.031
Clearance of *S*-hydroxynorketamine compartment 1; CLH1 (L/min @ 70 kg)	0.933	0.068	0.103	0.042	0.008	0.004
Clearance of *S*-hydroxynorketamine compartment 2; CLH2 (L/min @ 70 kg)	1.70	0.25	0.287	0.124		
Outlier (id = 9; occ = 2) CLH2 (L/min @ 70 kg)	0.36	0.02				
Mean transit time NK → HNK (min)	1.12	0.51				
σ_Relative_ (relative within subject variability)	0.079	0.005				
σ_Additive_ (additive within subject variability)	0.020	0.003				

*SEE, Standard error of estimate; K, S-ketamine; NK, S-norketamine; HNK, S-hydroxynorketamine*.

**Figure 4:**
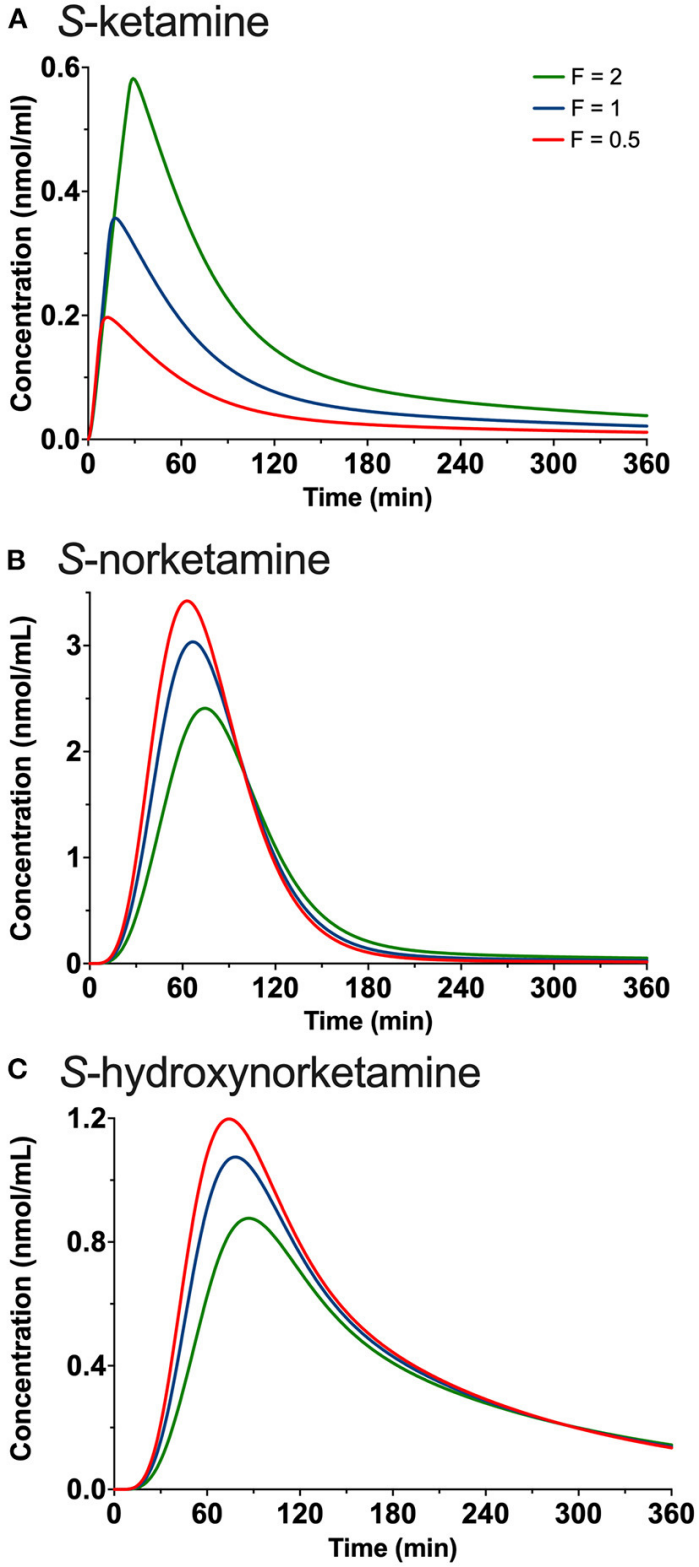
Simulations showing the effect of changing the duration of placement of the 50 mg oral thin file in the mouth by changing both F1 (bioavailability) and D (duration of absorption) on the plasma concentrations of *S*-ketamine **(A)**, *S*-norketamine **(B)** and *S*-hydroxynorketamine **(C)**. F is a factor by which D1 is adjusted and ranges from 0.5 (red lines) to 1 (blue lines) and 2 (green lines).

The bioavailability of *S*-ketamine from the OTF was 26.3 ± 1.0%, with a duration of absorption (D1) of 13 min and an absorption rate constant of 0.04 min^−1^ (KA1), with one outlier (subject #4) who had a KA1 value of 0.012 min^−1^. The bioavailability for the 50 mg and 100 mg OTF differed by about 20% (F1 50 mg = 29%, F1 100 mg = 23%) but this did not reach the level of significance (p ≈ 0.01). The *S*-ketamine that was not absorbed in the mouth was ingested and was absorbed in the remainder of the gastrointestinal system into the portal vein. This process was modeled by two delay compartments defined by an absorption rate constant KA2 and a mean transit time (MTTG, Figure 4). The gastrointestinal absorption (F2) took 30 min. Around 75% of the initial amount of *S*-ketamine was directly metabolized into S-norketamine without participating in the distribution of *S*-ketamine in the systemic circulation. Metabolism into *S*-norketamine was modeled by two delay compartments with the delay defined by two mean transit times (MTT K → NK, Table 2 and Figure 2), which has a population value of around 20 min (again with outlier subject #4 who had a value of 9 min). Twenty percent of *S*-ketamine was not metabolized into *S*-norketamine but was either metabolized into other metabolites (e.g., hydroxyketamine) or was lost in the gut. *S*-norketamine was metabolized into *S*-hydroxynorketamine *via* two metabolism compartments with the delay defined by two mean transit times (NK → HNK, Table 2 and Figure 2), which had a population value of around 1 min. Thirty percent of *S*-norketamine was not metabolized into *S*-hydroxynorketamine but was metabolized to other metabolites such as *S*-dehydronorketamine.

### Simulations

The results of the *in-silico* simulations are given in Figure 4. Increasing the duration of 50 mg oral thin film application in the mouth increased peak *S*-ketamine concentration by a factor of 2, while reducing the duration of the film in the mouth reduced peak *S*-ketamine concentration accordingly (Figure 4A). Both *S*-norketamine and *S*-hydroxynorketamine peak concentrations changed reciprocally to the changes in *S*-ketamine (Figures 4B,C) due to changes in the first-pass effect.

## Discussion

The main findings from our pharmacokinetic study on the *S*-ketamine oral thin film are summarized as follows: (i) the oral thin film was safe and the participants experienced mild adverse events infrequently related to the application of the film; (ii) *S*-ketamine bioavailability from the OTF was on average 26%; (iii) a 20% lower bioavailability of the 100 mg OTF relative to the 50 mg OTF was observed although this difference did not reach the level of significance; (iv) due to the large first pass-effect, 80% of *S*-ketamine was metabolized into *S*-norketamine leading to high concentrations of *S*-norketamine following sublingual or buccal film application for at least 6-h; (v) 56% of *S*-ketamine was finally metabolized into *S*-hydroxynorketamine, similarly, giving high plasma concentration for at least 6-h; (vi) no differences in pharmacokinetics were observed for the sublingual or buccal administration routes; (vii) pharmacokinetic parameter estimates are in agreement with earlier findings.

The OTF is rapidly, that is within 2 min, dissolved in saliva. Subjects were not allowed to swallow for 10 min after the oral film was applied, and retained the dissolved *S*-ketamine in their mouth. The process of local absorption took on average 13 min (Table 4), indicative that some *S*-ketamine remained on the mucosa after swallowing. The majority of the *S*-ketamine was swallowed after 10 min, and moved into the gastrointestinal tract, where it was absorbed and transported *via* the portal vein to the liver, where further biotransformation occurred. We remain uninformed regarding the 20% loss of *S*-ketamine. (2) This may be related to loss in the gut, or metabolism into other metabolites than *S*-norketamine. It is thought that about 10% of ketamine is eliminated unchanged in the gut. A minor metabolic pathway is the hydroxylation of *S*-ketamine into 4-hydroxyketamine and some other metabolites (e.g., hydroxyhpenylketamine) (12) The majority of *S*-ketamine (80%) undergoes hepatic *N*-demethylation into *S*-norketamine by cytochrome P450 (CYP) enzymes 2B6 and 3A4 (12, 13). We cannot exclude that some part of the *S*-ketamine is metabolized in extrahepatic tissues, such as oral or gut mucosal cells (14–16). This possibility is represented in the pharmacokinetic model (Figure 2) by the dotted red lines, which symbolize metabolic pathways of the oral and gut mucosa. Cytochrome P450 enzymes such as CYPA34 but not CYP2D6 are expressed in the oral mucosal lining (15). Similarly, the intestinal mucosa contains CYP3A4 and may possibly be an important route for first-pass conversion of *S*-ketamine and production of *S*-norketamine (15). However, previous studies showed just a minor role for gut wall clearance in the overall metabolism of *S*-ketamine with a ratio of intestinal mucosal clearance to hepatic clearance of 1:253. (17) Because of this reason and the fact that we cannot discriminate between first-pass hepatic clearance and gut wall clearance, we modeled the *S*-ketamine first-pass effect through parenchymal liver metabolism only. In the liver, *S*-norketamine is metabolized *via* cyclohexanone ring hydroxylation to form 4-, 5- and 6-hydroxynorketamine by CYP2B6 and CYP2A6 enzymes (12). A small amount of *S*-norketamine is dehydrogenated into dehydronorketamine by CYP2B6, while some dehydronorketamine may additionally be produced from S-hydroxynorketamine by dehydration (12). In the current analysis we just modeled the major metabolic pathways and assumed that 70% of *S*-norketamine was metabolized into *S*-hydroxynorketamine. This is based on earlier modeling studies that showed that a hydroxynorketamine to dehydronorketamine metabolic ratio of 70%:30% reflected best their measured plasma concentrations (9). Finally, all hydroxy products are glucuronidated in the liver and subsequently eliminated via bile and kidney (12).

Bioavailability of the oral thin film was on average 26% with a somewhat higher bioavailability for the 50 mg film than for the 100 mg film (F1 50 mg = 29%, F1 100 mg = 23%). Similar dose-dependency of bioavailability was observed for intranasal *S*-ketamine formulation that showed a decrease in bioavailability from 63% for a 28 mg *S*-ketamine dose to 50% for a 112 mg *S*-ketamine dose (18). Possibly a saturation in absorption is observed here. Alternatively, a longer absorption time by expanding the “do not swallow” period following film application would have increased F1 at the expense of the first-pass effect. In other words, *S*-ketamine bioavailability following OTF application is reciprocally related to the *S*-norketamine and *S*-hydroxynorketamine concentrations (Figure 4). This is also reflected in the ratio *S*-norketamine over *S*-ketamine. Earlier studies indicated that this ratio equals 5 following oral ketamine administration and 2 after sublingual application of a ketamine lozenge (19). In our study the ratio equals 4.6 after the 50 mg OTF and 5.1 after the 100 mg film. This and our model analysis indicate a large first-pass effect related to the transition of the *S*-ketamine into the gut after the ingestion of the remaining *S*-ketamine from the film after the 10-min “do not swallow” period and subsequently into the portal vein, or to metabolism directly in the mucosa of either the oral cavity or the remaining intestinal tract. As indicated above, we are unable to discriminate among these first-pass metabolic pathways. It is important to realize that depending on the clinical need, a large first-pass effect may be advantageous as it results in relatively high plasma concentrations of the ketamine metabolites. Particularly, high concentrations of hydroxynorketamine may be of interest when treating patients suffering from therapy-resistant depression (6). Figure 1 shows that OTF 50 and 100 mg *S*-hydroxynorketamine concentrations (as observed from t = 0 to 6 h) exceed the increase in *S*-hydroxynorketamine concentration from t = 6 to 8 h following the 20 mg intravenous *S*-ketamine infusion. To obtain similar concentration of *S*-hydroxynorketamine following intravenous *S*-ketamine administration would require much higher intravenous doses that would coincide with a higher probability of unwanted side effects (5). Whether hydroxynorketamine is analgesic in humans has not yet been tested as no hydroxynorketamine is available for human use. One animal study did find analgesic efficacy from (*2R,6R*)-hydroxynorketamine in several acute and chronic pain animal models. (20). In part 2 of our analysis, we performed a pharmacokinetic-pharmacodynamic analysis and took, apart from *S*-ketamine, both metabolites into account in the pharmacodynamic model. This (indirect) approach could not substantiate any effect of *S*-norketamine or *S*-hydroxynorketamine in the antinociceptive behavior of the *S*-ketamine OTF (4).

**Figure 5:**
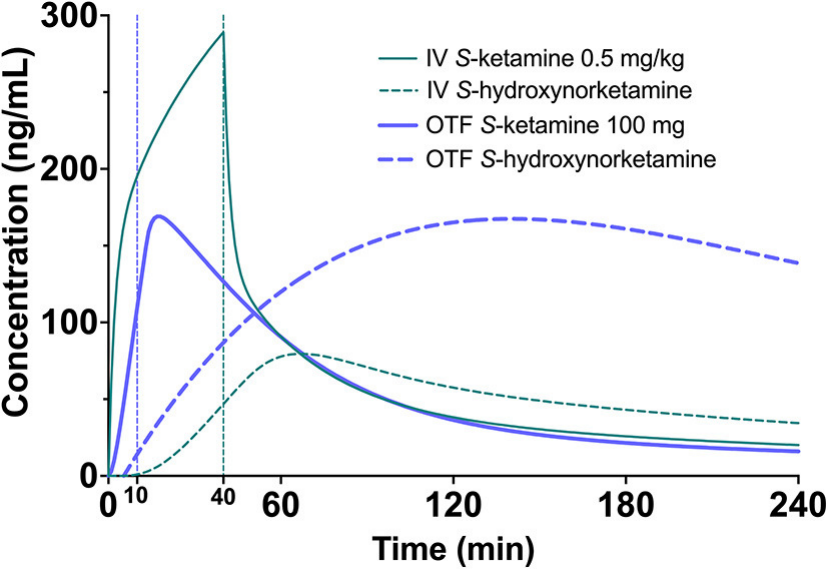
Simulation showing the *S*-ketamine (continuous green line) and *S*-hydroxynorketamine (broken green line) concentration profiles following a 0.5 mg/kg *S*-ketamine infusion, given for 40 min in a 70-kg individual. As comparator the equivalent concentrations are given following the 100 mg *S*-ketamine oral thin film (blue continuous = *S*-ketamine, and broken blue line = *S*-hydroxynorketamine).

The level of the sublingual/buccal *S*-ketamine bioavailability we observed fits well with earlier findings on sublingual ketamine formulations that ranged from 24 to 29% (2, 21) Bioavailability after oral administration is more variable and ranges from 8 to 24% (2, 3, 22, 23). A recent report on the population pharmacokinetics of *S*-ketamine nasal spray indicate a bioavailability of 54% from passage through the nasal cavity with about 19% of the swallowed dose reaching the systemic circulation (18). Finally, inhalation of *S*-ketamine has a bioavailability of 70% but is depending on the ketamine plasma concentration (8). At higher concentrations, due to sedation, ketamine is lost to the environment, and bioavailability decreases (at 275 and 375 ng/ml bioavailability is 50 and 38%, respectively). So, in comparison bioavailability for the different administration routes are oral < sublingual < intranasal < inhalation (albeit dose dependent) < intravenous administration. As indicated extending the sublingual or buccal absorption time of the OTF would likely have increased the *S*-ketamine concentration in plasma in our study (Figure 4). This may be an important consideration when treating acute pain with the OTF. Additionally, the *S*-ketamine oral thin film metabolic profile differs from other administration forms that exhibit a lesser first pass effect (including intravenous administration, Figure 5; the greater the first pass effect, the more norketamine and hydroxynorketamine is formed). This together with the differences in bioavailability will evidently affect the efficacy profile of the formulation for treatment of pain and depression.

Finally, since *S*-ketamine is an important treatment option for therapy-resistant depression,1 we simulated the *S*-ketamine and *S*-hydroxynorketamine profiles following 0.5 mg/kg intravenous ketamine given over 40 min to a 70 kg individual, which is the usual treatment dose for depression, and compared these profiles to those observed after the 100 mg *S*-ketamine oral thin film. The results indicate greater *S*-ketamine concentrations after the intravenous infusion but lower *S*-hydroxynorketamine concentrations compared to the oral thin film (Figure 5). Since the role of the various ketamine metabolites such as hydroxynorketamine remain unknown in producing the antidepressant effects of ketamine (1, 24), a study on the effect of the *S*-ketamine oral thin film in patients with depression may shed light on this matter.

In conclusion, the *S*-ketamine oral thin film is a safe and practical alternative to intravenous *S*-ketamine administration that results in relatively high concentrations of *S*-ketamine and its two metabolites.

## Data Availability Statement

The data are available from the authors after agreement has been obtained regarding purpose of analysis and protocol.

## Ethics Statement

The studies involving human participants were reviewed and approved by METC-LDD. The patients/participants provided their written informed consent to participate in this study.

## Author Contributions

PS, AD, MV, MN, PM, and FH were involved in the design of the study. PS, ML, TD, MN, RS, and AD performed experiments and contributed to data collected. AD and EO designed the statistical analysis and performed data analysis. MN and MV supervised the project. RM performed the chemical analysis. AD wrote the initial draft of the manuscript. All authors were contributed to data interpretation, final drafting of the manuscript, and approved the submitted version.

## Funding

This study was supported by LTS Lohmann Therapie-Systeme AG, Andernach, Germany and institutional funds. LTS Lohmann Therapie-Systeme AG was not involved in the study design, collection, analysis, interpretation of data, the writing of this article or the decision to submit it for publication.

## Conflict of Interest

PM and FH are employees of LTS Lohmann Therapie-Systeme AG, Andernach, Germany. The remaining authors declare that the research was conducted in the absence of any commercial or financial relationships that could be construed as a potential conflict of interest.

## Publisher's Note

All claims expressed in this article are solely those of the authors and do not necessarily represent those of their affiliated organizations, or those of the publisher, the editors and the reviewers. Any product that may be evaluated in this article, or claim that may be made by its manufacturer, is not guaranteed or endorsed by the publisher.
