# Supplementary material for: S-Ketamine Oral Thin Film—Part 1: Population Pharmacokinetics of S-Ketamine, S-Norketamine and S-Hydroxynorketamine
Source: Front Pain Res (Lausanne). 2022 Jul 11;3:946486. doi: 10.3389/fpain.2022.946486 (PMC9309697; doi:10.3389/fpain.2022.946486)
Supplement: Supplementary file 1 [file Data_Sheet_1.PDF]

Supplemental materials

to

S-ketamine oral thin film – Part 1: population pharmacokinetics of *S*-ketamine, *S*-norketamine and *S*-hydroxynorketamine

Pieter Simons, Erik Olofsen, Monique van Velzen, Maarten van Lemmen, René Mooren, Tom van Dasselaar, Patrick Mohr, Florian Hammes, Rutger van der Schrier Marieke Niesters, Albert Dahan

Supplemental Figure 1: individual pharmacokinetic data fits of *S*-ketamine, *S*-norketamine and *S*-hydroxynorketamine following the 50 mg and 100 mg S-ketamine oral thin films. The numbers are subject identification numbers.

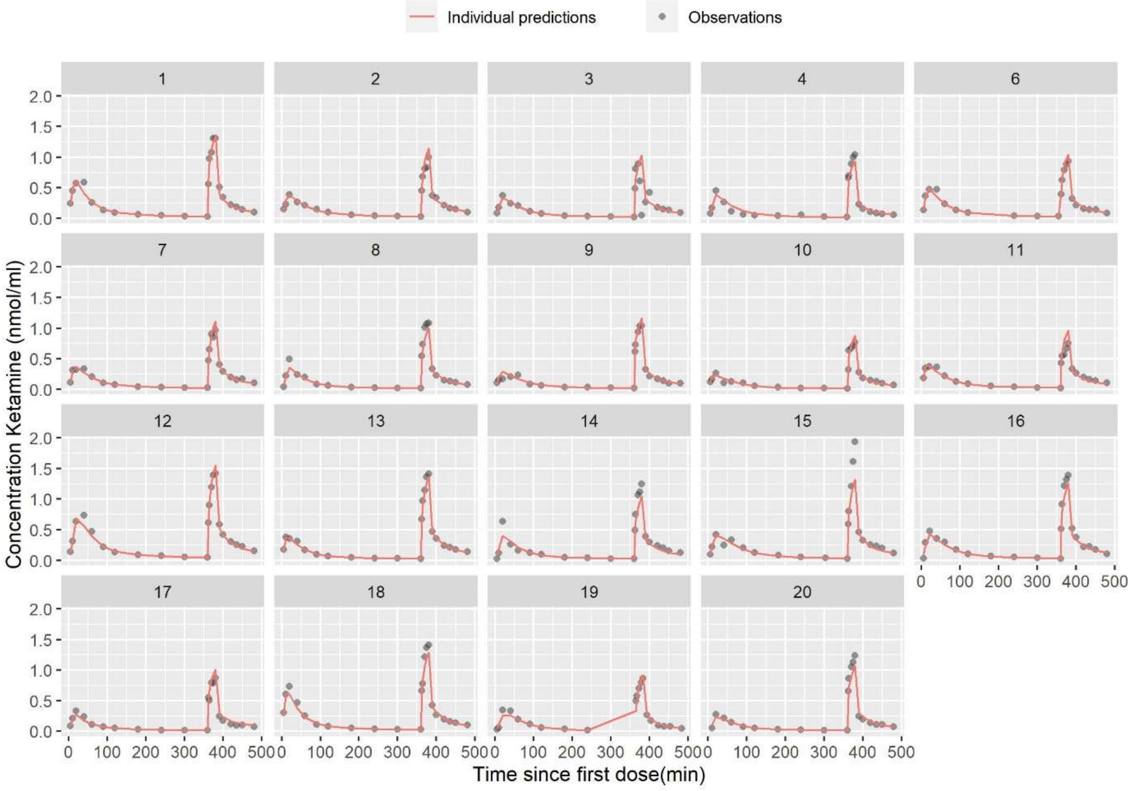

Figure 1S. A. *S*-ketamine oral thin film 50 mg: *S*-ketamine data fits.
R^2-values are:

Subject 1	Subject 2	Subject 3	Subject 4	Subject 6
0.961	0.942	0.147	0.968	0.972
Subject 7	Subject 8	Subject 9	Subject 10	Subject 11
0.964	0.960	0.835	0.962	0.825
Subject 12	Subject 13	Subject 14	Subject 15	Subject 16
0.982	0.582	0.884	0.955	0.969
Subject 17	Subject 18	Subject 19	Subject 20	
0.919	0.963	0.962	0.957	

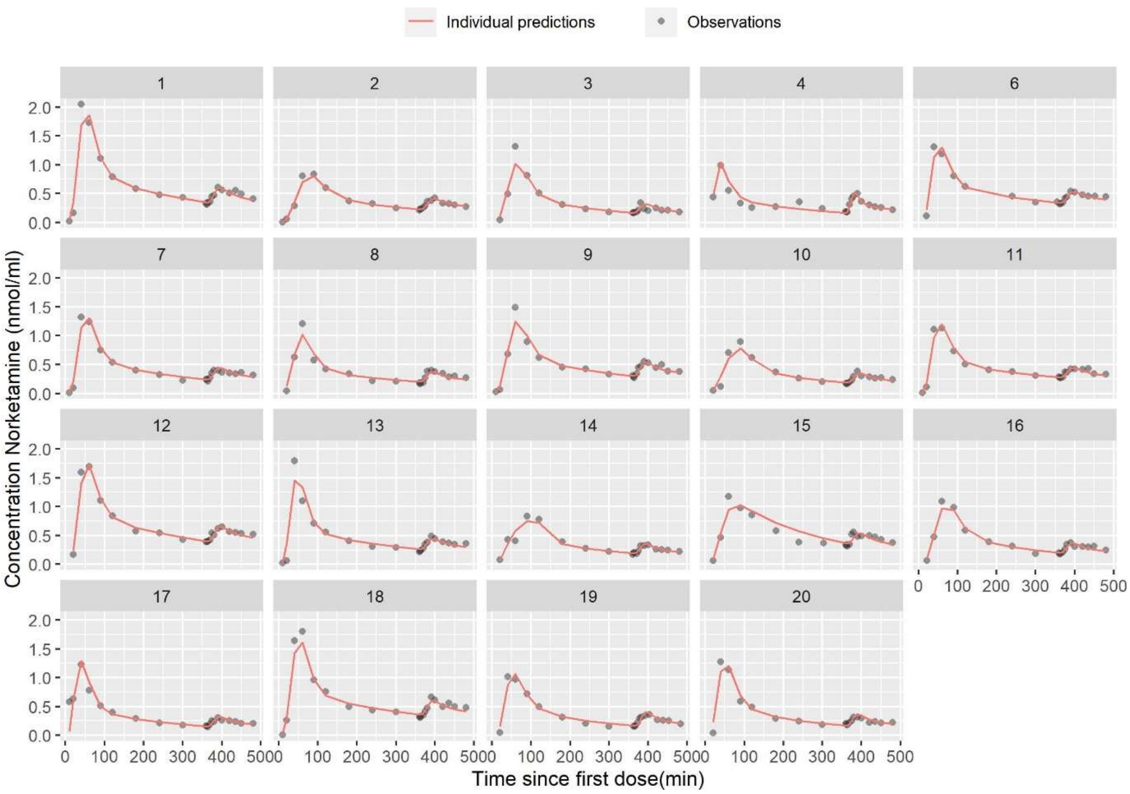

Figure 1 S. B. *S*-ketamine oral thin film 50 mg: *S*-norketamine data fits

R^2-values are:

Subject 1	Subject 2	Subject 3	Subject 4	Subject 6
0.937	0.972	0.979	0.894	0.938
Subject 7	Subject 8	Subject 9	Subject 10	Subject 11
0.965	0.934	0.673	0.925	0.972
Subject 12	Subject 13	Subject 14	Subject 15	Subject 16
0.978	0.905	0.989	0.962	0.972
Subject 17	Subject 18	Subject 19	Subject 20	
0.963	0.986	0.958	0.949	

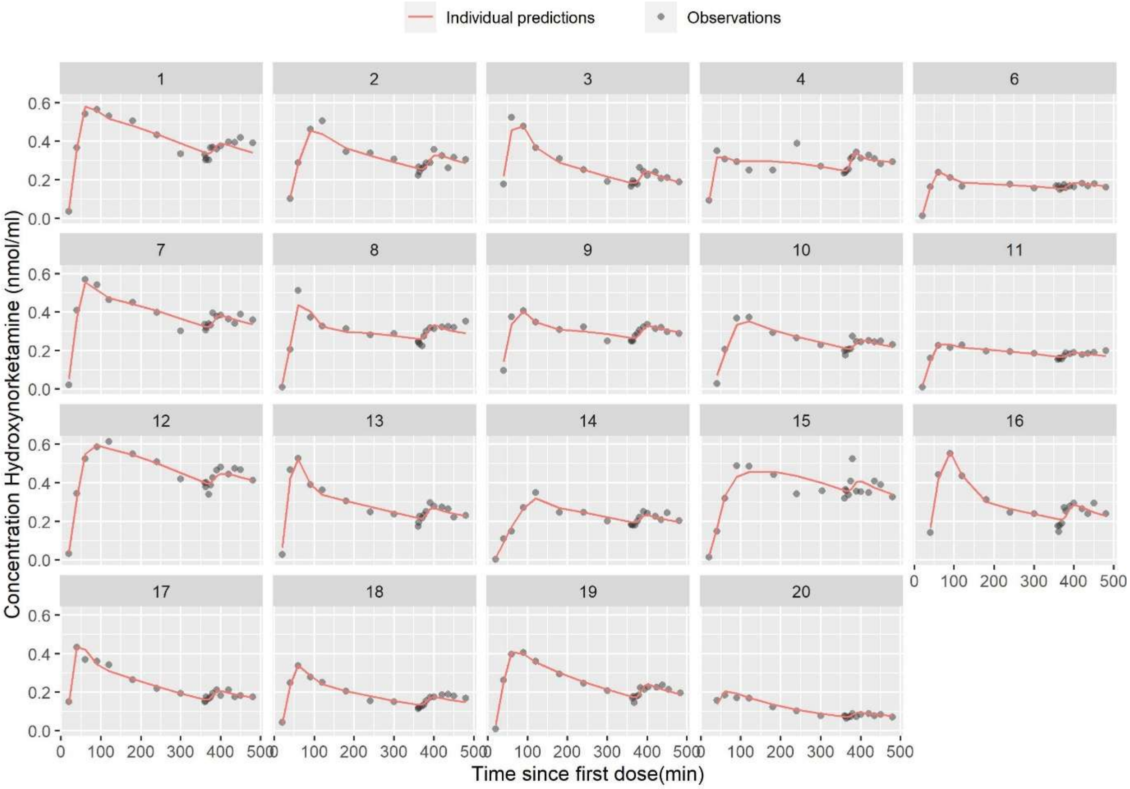

Figure 1S. C. *S*-ketamine oral thin film 50 mg: *S*-hydroxynorketamine data fits
R^2-values are:

Subject 1	Subject 2	Subject 3	Subject 4	Subject 6
0.929	0.981	0.933	0.894	0.936
Subject 7	Subject 8	Subject 9	Subject 10	Subject 11
0.769	0.880	0.901	0.939	0.925
Subject 12	Subject 13	Subject 14	Subject 15	Subject 16
0.910	0.913	0.936	0.790	0.982
Subject 17	Subject 18	Subject 19	Subject 20	
0.956	0.950	0.859	0.659	

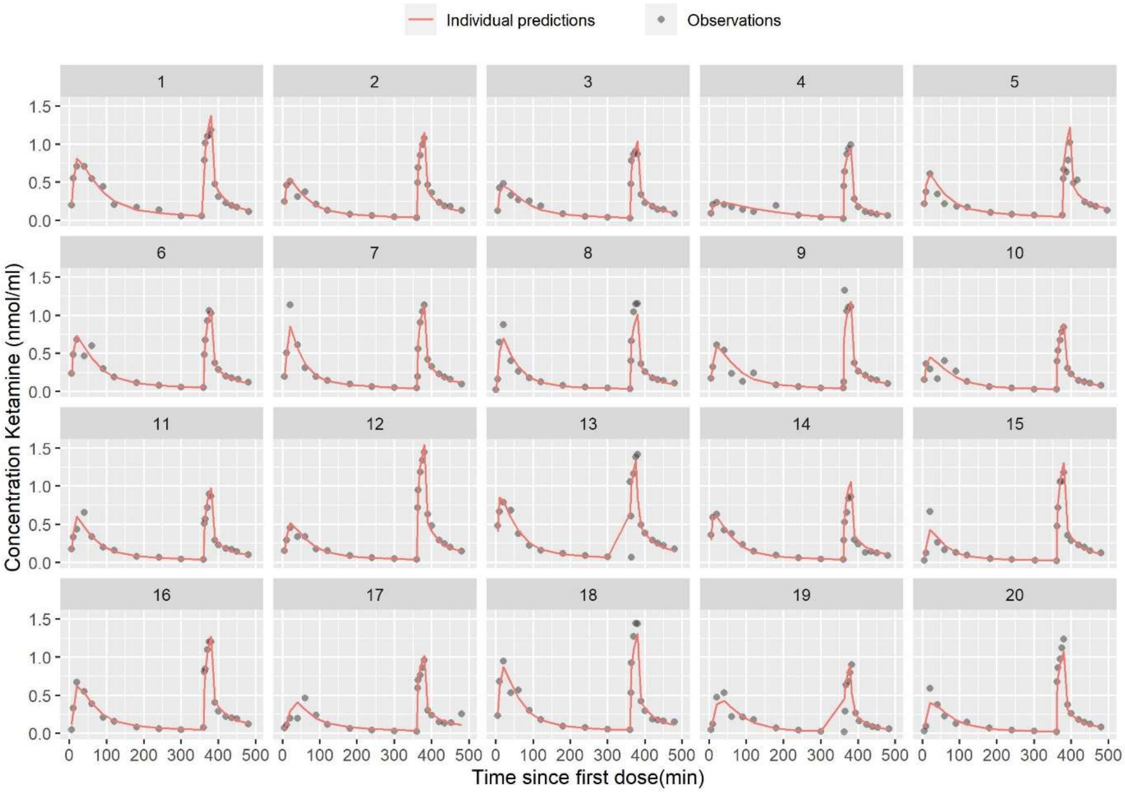

Figure 1S. D. *S*-ketamine oral thin film 100 mg: *S*-ketamine data fits
R^2-values are:

Subject 1	Subject 2	Subject 3	Subject 4	Subject 5
0.979	0.972	0.964	0.983	0.746
Subject 6	Subject 7	Subject 8	Subject 9	Subject 10
0.969	0.945	0.941	0.980	0.920
Subject 11	Subject 12	Subject 13	Subject 14	Subject 15
0.934	0.983	0.993	0.930	0.895
Subject 16	Subject 17	Subject 18	Subject 19	Subject 20
0.975	0.939	0.974	0.685	0.964

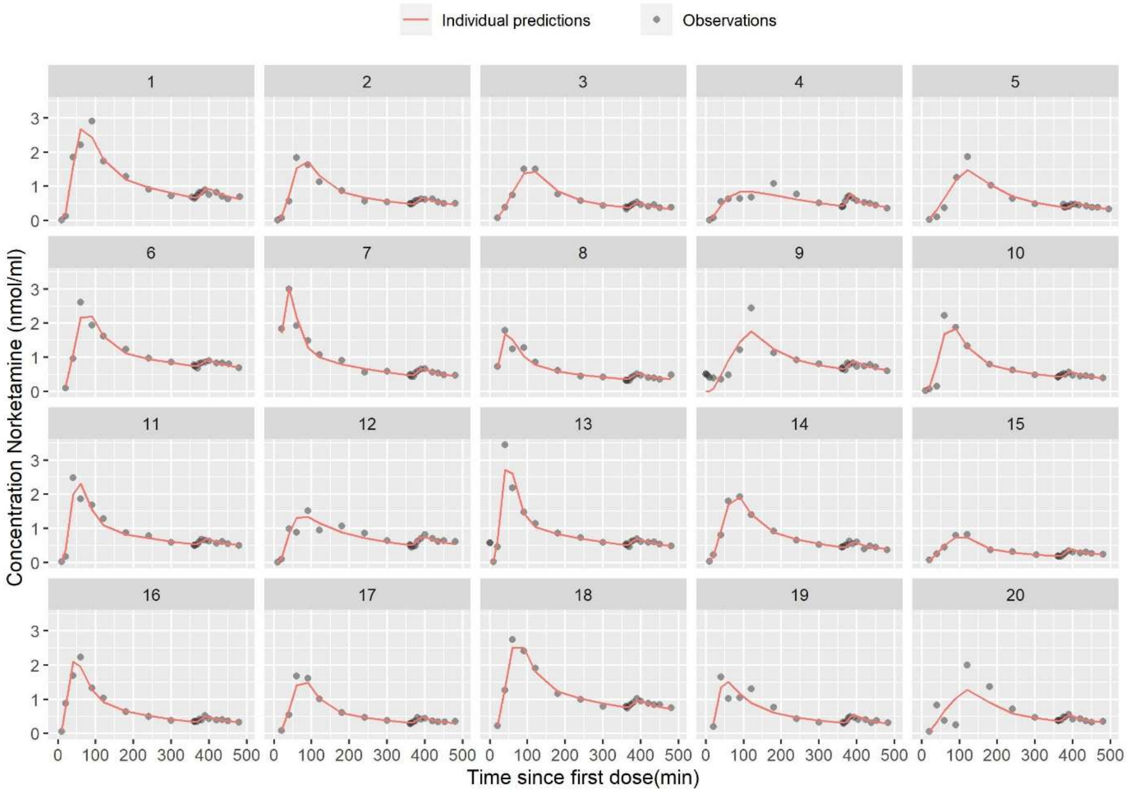

Figure 1S. E. *S*-ketamine oral thin film 100 mg: *S*-norketamine data fits
R^2-values are:

Subject 1	Subject 2	Subject 3	Subject 4	Subject 5
0.958	0.944	0.918	0.784	0.907
Subject 6	Subject 7	Subject 8	Subject 9	Subject 10
0.957	0.982	0.938	0.950	0.872
Subject 11	Subject 12	Subject 13	Subject 14	Subject 15
0.914	0.824	0.905	0.923	0.858
Subject 16	Subject 17	Subject 18	Subject 19	Subject 20
0.947	0.796	0.968	0.799	0.520

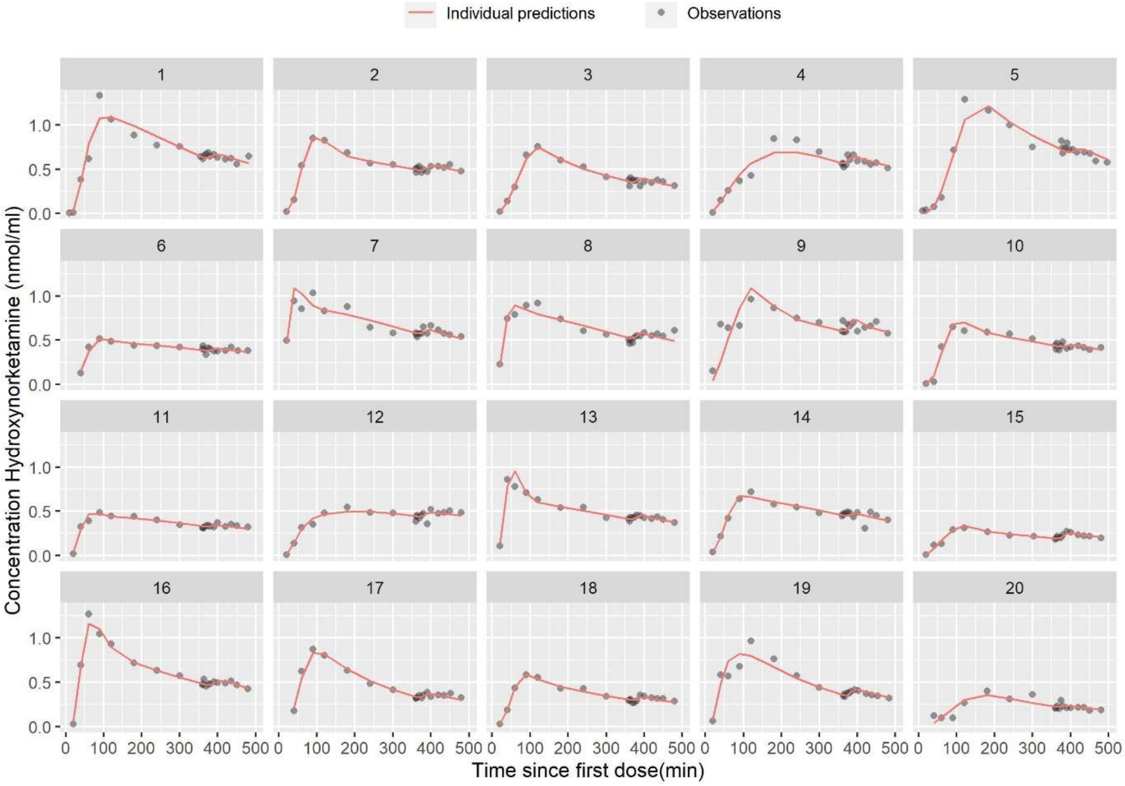

Figure 1S. F. *S*-ketamine oral thin film 100 mg: *S*-hydroxynorketamine data fits
R^2-values are:

Subject 1	Subject 2	Subject 3	Subject 4	Subject 5
0.916	0.910	0.961	0.753	0.948
Subject 6	Subject 7	Subject 8	Subject 9	Subject 10
0.889	0.942	0.906	0.712	0.900
Subject 11	Subject 12	Subject 13	Subject 14	Subject 15
0.939	0.954	0.913	0.903	0.938
Subject 16	Subject 17	Subject 18	Subject 19	Subject 20
0.924	0.970	0.976	0.977	0.659
